# Reduced B7-H3 expression by *PAX3-FOXO1* knockdown inhibits cellular motility and promotes myogenic differentiation in alveolar rhabdomyosarcoma

**DOI:** 10.1038/s41598-021-98322-z

**Published:** 2021-09-22

**Authors:** Takuyo Kanayama, Mitsuru Miyachi, Yohei Sugimoto, Shigeki Yagyu, Ken Kikuchi, Kunihiko Tsuchiya, Tomoko Iehara, Hajime Hosoi

**Affiliations:** Department of Pediatrics, Graduate School of Medical Science, Kyoto Prefectural University of Medicine, 465, Kajii-cho, Kawaramachi-Hirokoji, Kamigyo-ku, Kyoto, 602-8566 Japan

**Keywords:** Cancer, Oncology

## Abstract

B7-H3 (also known as CD276) is associated with aggressive characteristics in various cancers. Meanwhile, in alveolar rhabdomyosarcoma (ARMS), PAX3-FOXO1 fusion protein is associated with increased aggressiveness and poor prognosis. In the present study, we explored the relationship between PAX3-FOXO1 and B7-H3 and the biological roles of B7-H3 in ARMS. Quantitative real time PCR and flow cytometry revealed that *PAX3-FOXO1* knockdown downregulated B7-H3 expression in all the selected cell lines (Rh-30, Rh-41, and Rh-28), suggesting that PAX3-FOXO1 positively regulates B7-H3 expression. Gene expression analysis revealed that various genes and pathways involved in chemotaxis, INF-γ production, and myogenic differentiation were commonly affected by the knockdown of *PAX3-FOXO1* and *B7-H3*. Wound healing and transwell migration assays revealed that both PAX3-FOXO1 and B7-H3 were associated with cell migration. Furthermore, knockdown of *PAX3-FOXO1* or *B7-H3* induced myogenin expression in all cell lines, although myosin heavy chain induction varied depending on the cellular context. Our results indicate that PAX3-FOXO1 regulates B7-H3 expression and that PAX3-FOXO1 and B7-H3 are commonly associated with multiple pathways related to an aggressive phenotype in ARMS, such as cell migration and myogenic differentiation block.

## Background

B7-H3 (also known as CD276), a member of the B7 family of type I transmembrane proteins, is known to modulate T-cell function in a costimulatory^1,2^ or coinhibitory^3,4^ manner. Although B7-H3 protein has limited expression in normal human tissues, it is broadly overexpressed in various human cancers ^5–9^, including rhabdomyosarcoma ^10^. Therefore, B7-H3 has been highlighted in recent years as an attractive surface antigen for molecular targeted therapy, such as those using monoclonal antibody ^11^ and chimeric antigen receptor (CAR) T-cells^10,12^. In addition to its immune regulatory roles in the tumor microenvironment, B7-H3 is known to be associated with tumor cell proliferation, migration, invasion, metabolism, and angiogenesis; therefore, it is related to a poor prognosis^13–19^.

Rhabdomyosarcoma (RMS) is the most common soft tissue malignancy in children^20^. It consists of two major subtypes, namely the alveolar RMS (ARMS) and the embryonal RMS (ERMS). The majority of ARMS are associated with specific fusion proteins, PAX3-FOXO1 or PAX7-FOXO1^21–23^. The expression of PAX3-FOXO1 is associated with increased aggressiveness and a poor prognosis^24^. PAX3-FOXO1 may function as a more potent transcriptional activator than PAX3, and induces the expression of a number of transcriptional targets that lead to tumorigenesis, cell proliferation, migration, invasion, and differentiation block^25–27^.

Although PAX3-FOXO1 contributes to aggressive characteristics in ARMS, and B7-H3 does the same in other cancers, limited information is available regarding the function of B7-H3 in ARMS and about the relationship between PAX3-FOXO1 and B7-H3. In this context, we hypothesized that PAX3-FOXO1 regulates B7-H3 expression and contributes to aggressive characteristics in ARMS. In this study, we analyzed the association of PAX3-FOXO1 and B7-H3 by knocking down the expression of *PAX3-FOXO1* and performing gene expression analysis. We demonstrate that *PAX3-FOXO1* knockdown downregulates the expression of B7-H3 in ARMS and that PAX3-FOXO1 and B7-H3 share common gene expression profiles, which contribute to the aggressive phenotype of ARMS.

## Results

### B7-H3 expression is downregulated by *PAX3-FOXO1* knockdown in alveolar rhabdomyosarcoma

**Figure 1:**
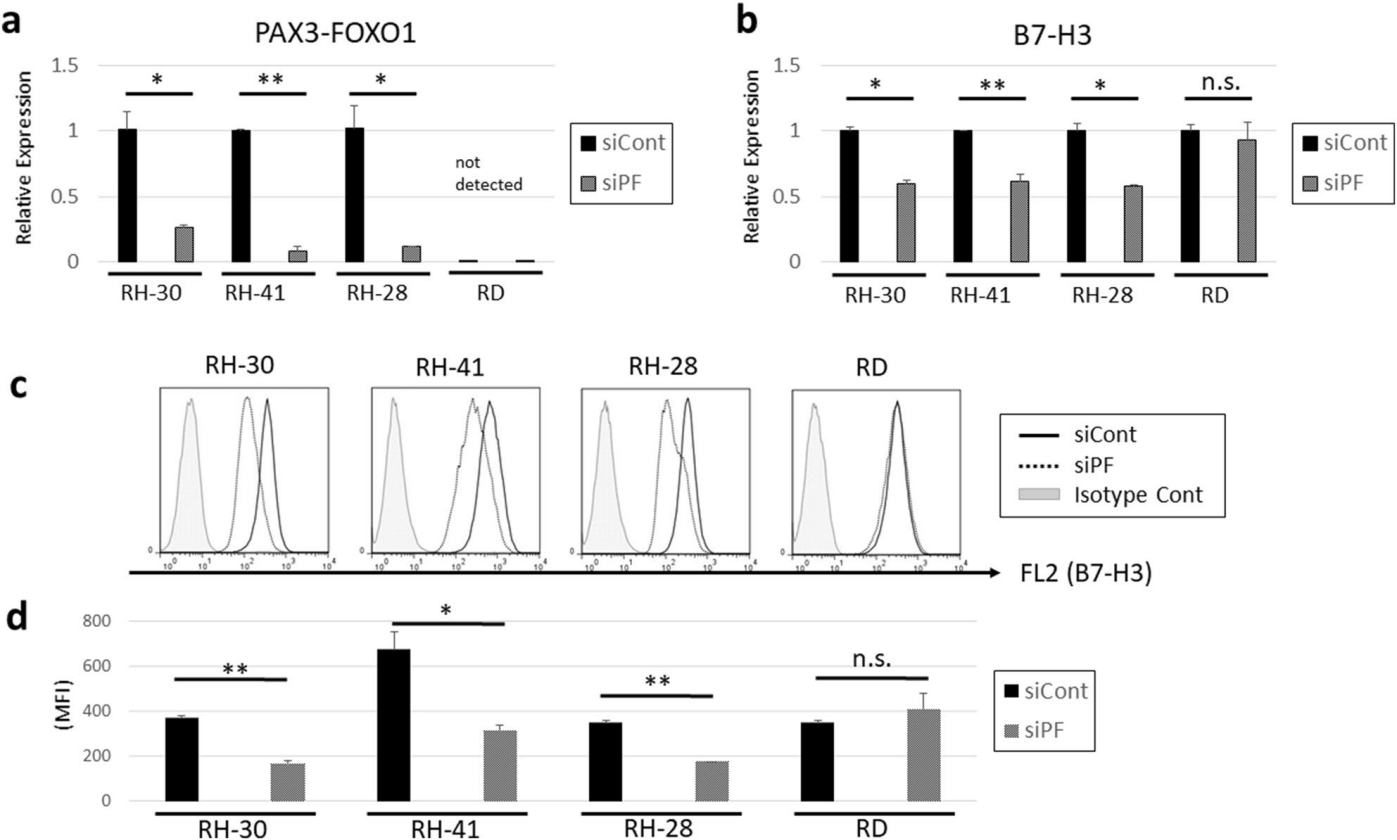
Knockdown of *PAX3-FOXO1* downregulates the expression of *B7-H3*. (**a**) Quantitative real-time PCR (qRT-PCR) analysis of *PAX3-FOXO1*. Knockdown efficacy of *PAX3-FOXO1* was confirmed in three *PAX3-FOXO1* positive ARMS cell lines. RD, a *PAX3-FOXO1*-negative cell line, was used as a negative control. (**b**) qRT-PCR analysis of *B7-H3*. (**c**) Representative histogram of flow cytometry analysis of B7-H3. (**d**) Mean fluorescence index of B7-H3 in flow cytometry analysis. Results shown are means ± SD. *p < 0.05, **p < 0.01, n.s. *p* > 0.05, by two-tailed unpaired *t* test.

To investigate the effect of *PAX3-FOXO1* on the expression of B7-H3, knockdown of *PAX3-FOXO1* transcripts was performed by siRNA targeting *PAX3-FOXO1* (siPF). In each *PAX3-FOXO1* fusion gene-positive ARMS cell line (RH-30, RH-41, and RH-28), a high knockdown efficiency of *PAX3-FOXO1* was confirmed by quantitative real time polymerase chain reaction (qRT-PCR), although *PAX3-FOXO1* was not detected in *PAX3-FOXO1*-negative RD cells (Fig. 1a). Next, qRT-PCR for *B7-H3* revealed that *PAX3-FOXO1* knockdown decreased the expression of *B7-H3* in the *PAX3-FOXO1*-positive cell line, but not in the *PAX3-FOXO1-*negative RD cells (Fig. 1b). In addition, flow cytometry analysis demonstrated that *PAX3-FOXO1* knockdown decreased the expression of B7-H3 in the *PAX3-FOXO1*-positive cell lines, but not in the *PAX3-FOXO1-*negative RD cells (Fig. 1c,d). These results suggest that PAX3-FOXO1 positively regulates the expression of B7-H3 at both the mRNA and protein levels.

### PAX3-FOXO1 and B7-H3 are associated with multiple pathways related to an aggressive rhabdomyosarcoma phenotype

**Figure 2:**
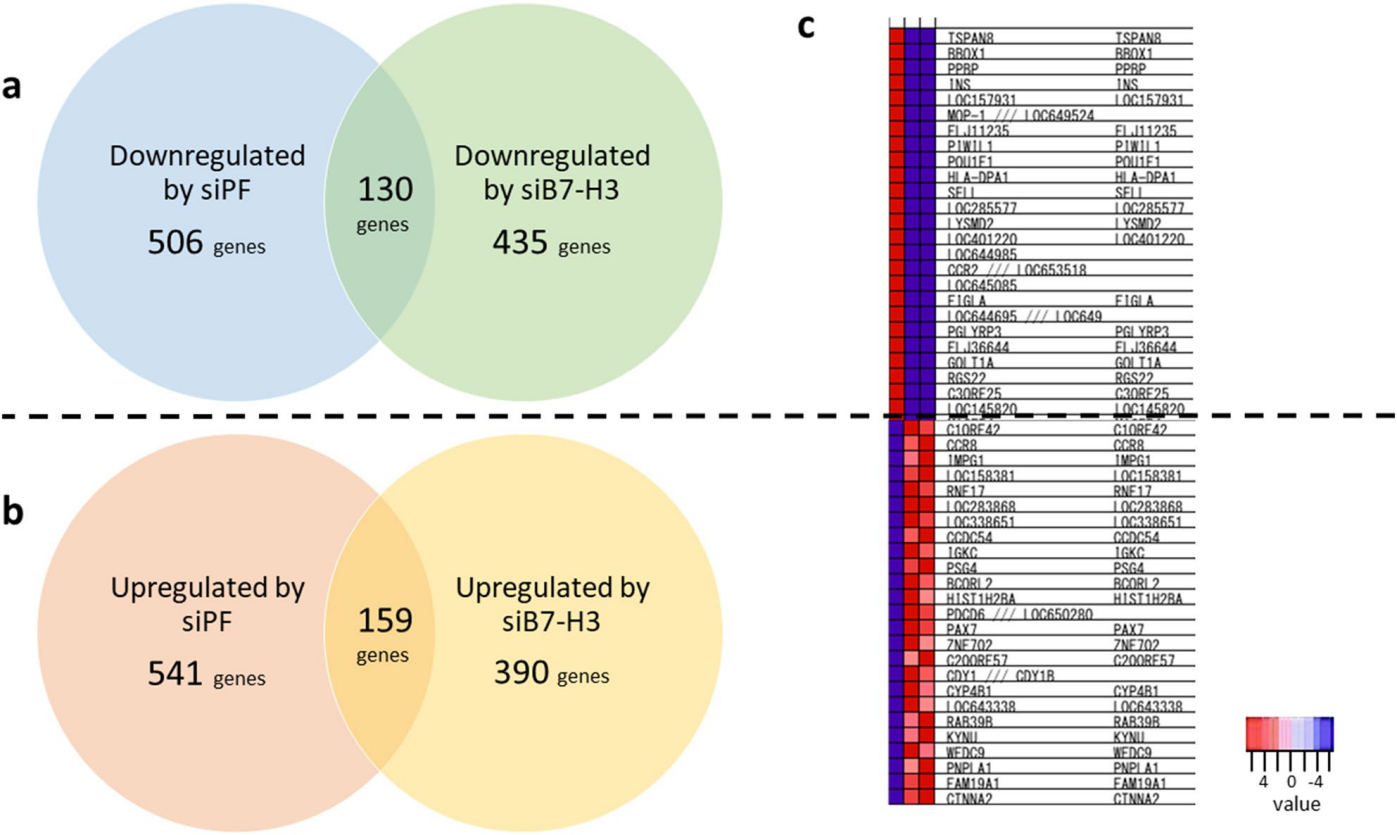
Gene expression analysis. Knockdown of *PAX3-FOXO1* and *B7-H3* reveal shared down/upregulated genes. (**a**) Venn diagram of genes that were downregulated by the knockdown of *PAX3-FOXO1* or *B7-H3*. (**b**) Venn diagram of genes that were upregulated by the knockdown of *PAX3-FOXO1* or *B7-H3*. (**c**) Heat map of the top 50 genes that were down/up-regulated by the knockdown of *PAX3-FOXO1* or *B7-H3*. The left line shows Rh-30 siCont, the middle Rh-30 siPF, and the right siB7-H3.

To unravel the pathways affected by *PAX3-FOXO1* and *B7-H3* in ARMS, gene expression analysis was performed using microarray and gene set enrichment analysis (GSEA). The gene expression signature of siPF or siRNA targeting B7-H3 (siB7-H3) transfected Rh-30 cells (Rh-30 siPF or Rh-30 siB7-H3, respectively) was compared with that of control siRNA (siCont) transfected Rh-30 cells (Rh-30 siCont). Gene expression analysis revealed that in Rh-30 siPF and Rh-30 siB7-H3, the expression levels of 636 and 565 genes were decreased, respectively (log 2 ratio, ≤ 1.0), and those of 700 and 549 genes were increased, respectively (log 2 ratio, ≥ 1.0), compared with the respective levels in Rh-30 siCont (Fig. 2a,b). Among them, the expression levels of 130 genes were downregulated and those of 159 genes were upregulated in both the Rh-30 siPF and Rh-30 siB7-H3 compared to the respective levels in the Rh-30 siCont (Fig. 2a,b). A heat map of top 50 shared downregulated or upregulated genes is shown in Fig. 2c.

**Figure 3:**
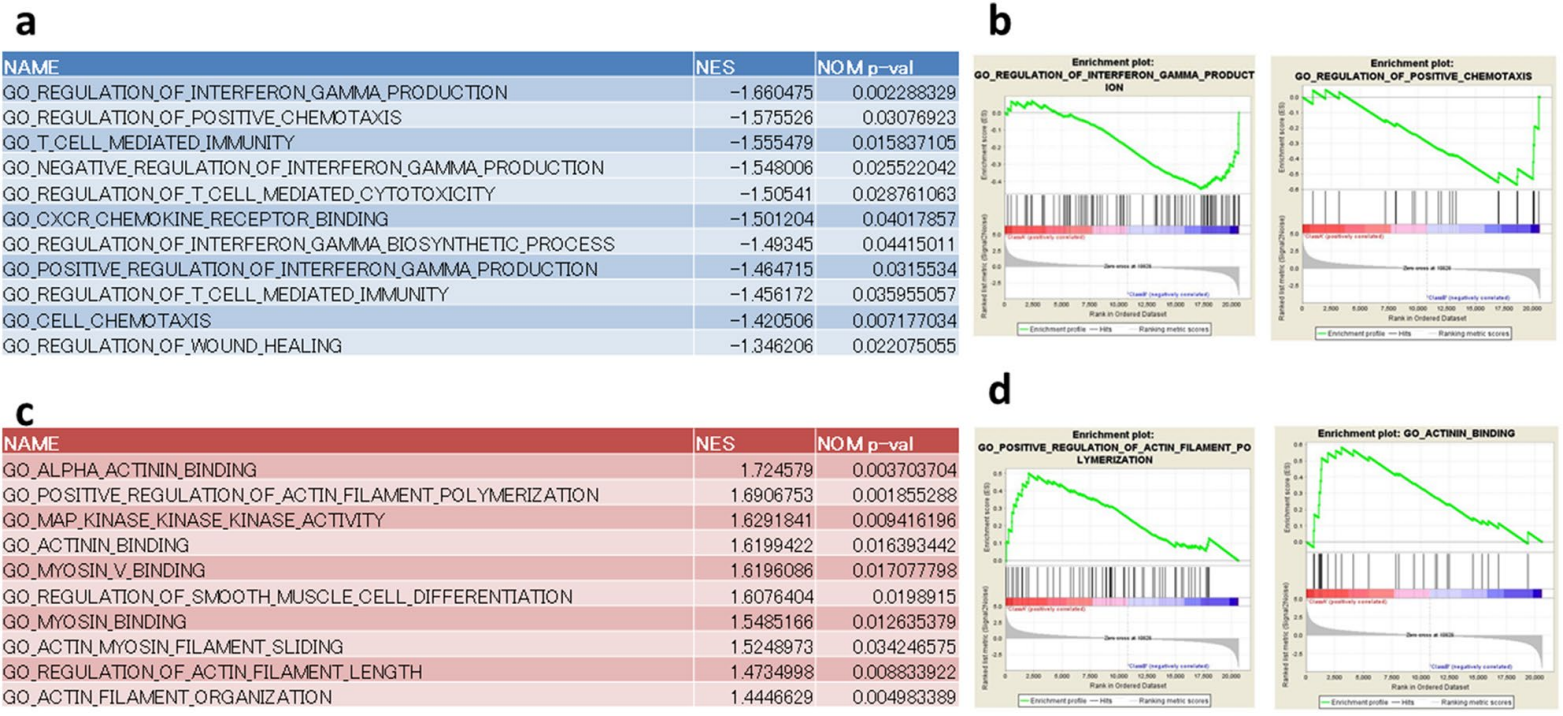
Gene set enrichment analysis (GSEA). (**a**) Representative list of impoverished gene sets upon knockdown of *PAX3-FOXO1* and *B7-H3*. (**b**) Enrichment plot from the GSEA. Interferon gamma production (left panel) and chemotaxis (right panel). (**c**) Representative list of enriched gene sets upon knockdown of *PAX3-FOXO1* and *B7-H3*. (**d**) Enrichment plot from the GSEA. Actin filament polymerization (left panel) and actin binding (right panel).

GSEA revealed that several pathways related to chemotaxis, INF-γ production, and regulation of T cell immunity were inactivated in both Rh-30 siPF and Rh-30 siB7-H3 unlike in Rh30 siCont, suggesting that *PAX3-FOXO1* and *B7-H3* may contribute to tumor cell metastasis and immune evasion (Fig. 3a,b). In contrast, several pathways related to muscle cell differentiation were activated in both Rh-30 siPF and Rh-30 siB7-H3 but not in Rh30 siCont, suggesting that *PAX3-FOXO1* and *B7-H3* may block myoblast differentiation (Fig. 3c,d).

### PAX3-FOXO1 and B7-H3 are associated with cell migration

**Figure 4:**
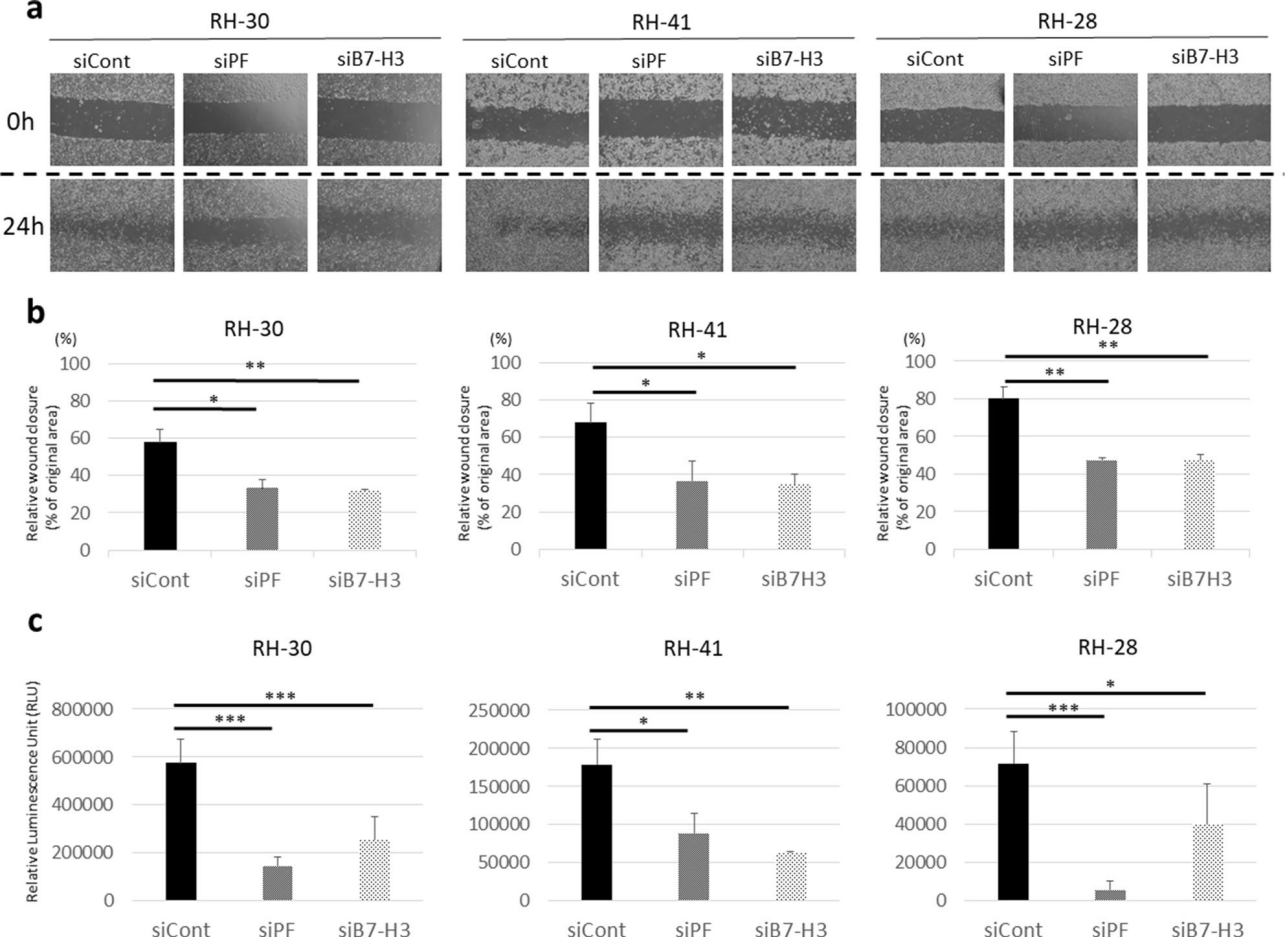
Knockdown of *PAX3-FOXO1* or *B7-H3* attenuates cell migration. (**a**) Representative figure of a wound healing assay. (**b**) Relative healed wound area (% of original wound area) in a wound healing assay. Knockdown of *PAX3-FOXO1* or *B7-H3* attenuates cell migration. (**c**) Transwell assay. Relative luminescence units are shown as the quantification of migratory cells. Results shown are means ± SD. *p < 0.05, **p < 0.01, ***p < 0.001, by two-tailed unpaired *t* test.

To confirm that *PAX3-FOXO1* and *B7-H3* contribute to metastasis in ARMS, as suggested by GSEA, we performed wound healing and transwell assays. Wound healing assays revealed that cell migration in ARMS was significantly attenuated by the knockdown of *PAX3-FOXO1* or *B7-H3* in all the three cell lines (Fig. 4a,b). The transwell assays similarly demonstrated that cell migration in ARMS was significantly attenuated by the knockdown of *PAX3-FOXO1* or *B7-H3* in all the three cell lines (Fig. 4c).

**Figure 5:**
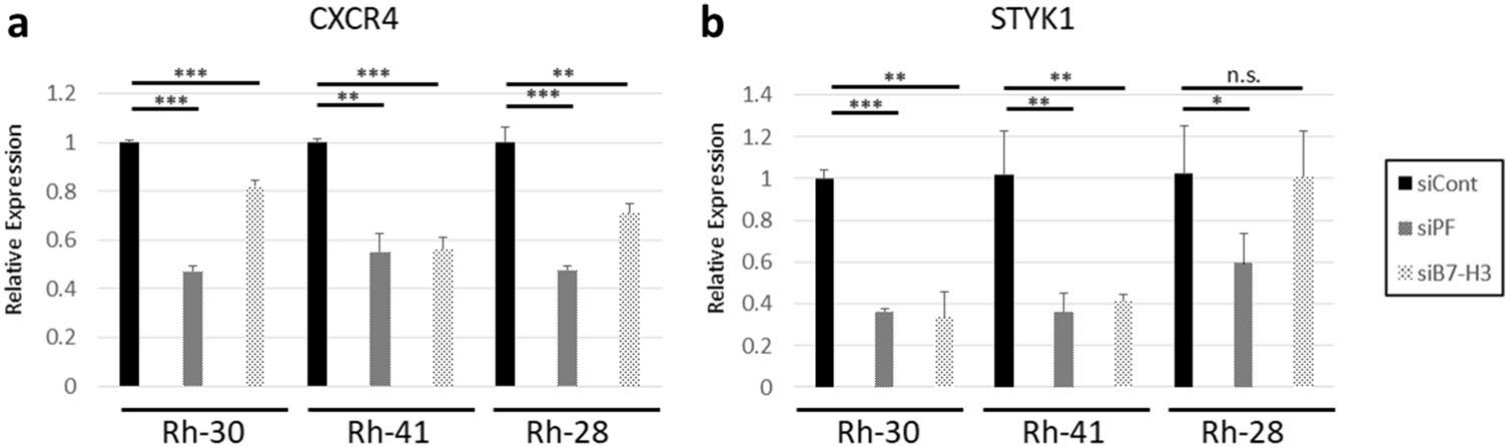
*CXCR4* and *STYK1* expression levels are regulated by PAX3-FOXO1 and B7-H3. (**a**) Quantitative real-time PCR (qRT-PCR) analysis of *CXCR4*. (**b**) qRT-PCR analysis of *STYK1*. Results shown represent means ± SD. *p < 0.05, **p < 0.01, ***p < 0.001, n.s. *p* > 0.05, by two-tailed unpaired *t* test.

To determine the pathways contributing to cell migration that are affected by *PAX3-FOXO1* and *B7-H3*, we performed qRT-PCR for *CXCR4* and *STYK1*, as gene expression analysis indicated that both these genes were downregulated in Rh-30 siPF and Rh-30 siB7-H3. As expected, *CXCR4* was downregulated by transfection of all the three cell lines with siPF or siB7-H3 (Fig. 5a). In addition, *STYK1* was downregulated in Rh-30 and Rh-41 by transfection with siPF or siB7-H3, although in Rh28, *STYK1* was downregulated by transfection with siPAX3-FOXO1, but not siB7-H3 (Fig. 5b).

### PAX3-FOXO1 and B7-H3 negatively regulate myogenin expression and interfere with myogenic differentiation

**Figure 6:**
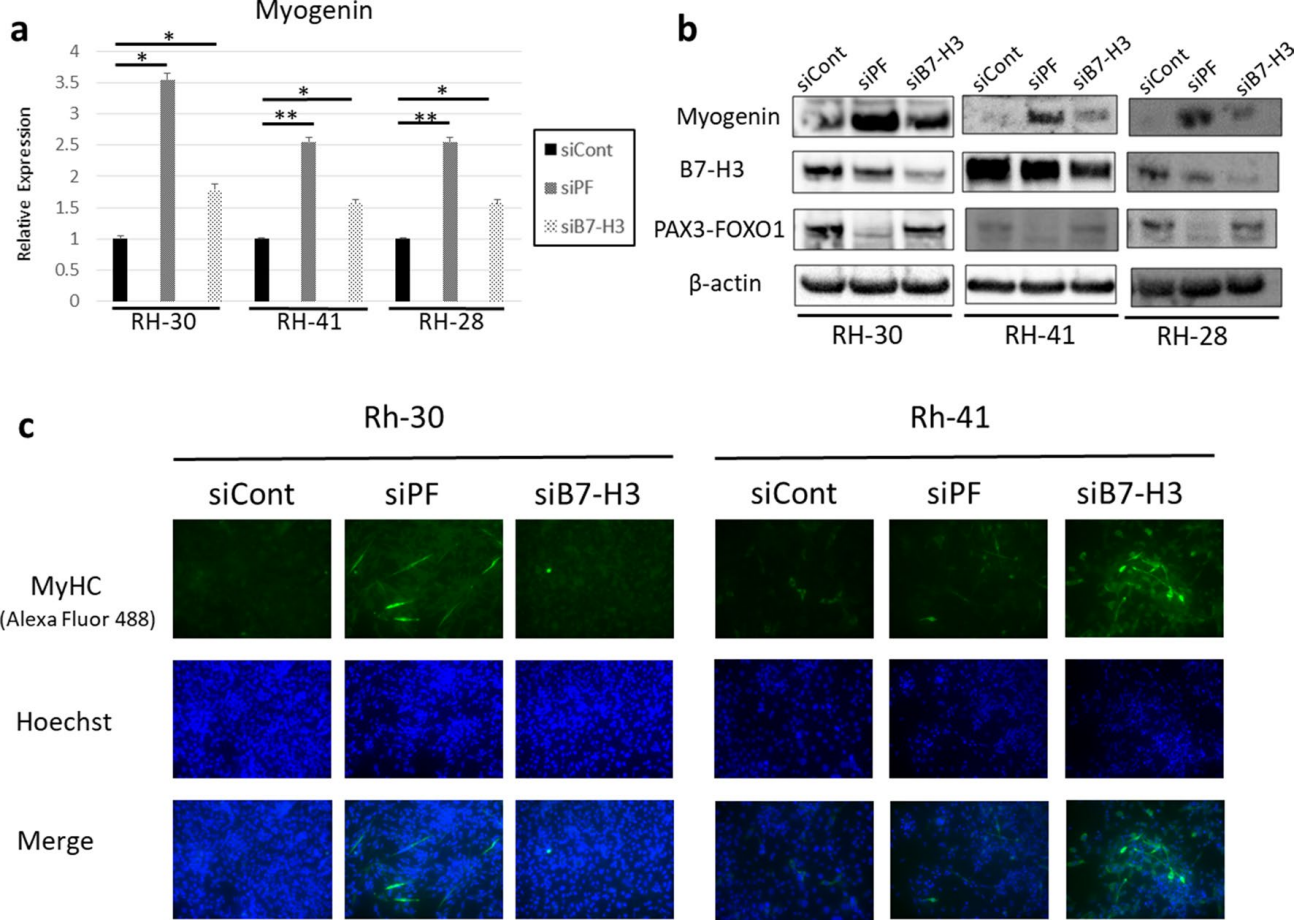
Knockdown of *PAX3-FOXO1* and *B7-H3* induces the expression of myogenin and may induce myoblast differentiation. (**a**) Quantitative real-time PCR analysis of *myogenin*. (**b**) Western blot analysis of myogenin. Knockdown of *PAX3-FOXO1* (detected by anti-PAX3 antibody) and *B7-H3* induces the expression of myogenin. (**c**) Immunofluorescence of myosin heavy chain. Results shown represent means ± SD. *p < 0.05, **p < 0.01, by two-tailed unpaired *t* test.

Because the results of GSEA suggested that PAX3-FOXO1 and B7-H3 may block myoblast differentiation, we investigated the expression level of myogenin, a transcription factor required for myoblast differentiation, following knockdown of *PAX3-FOXO1* or *B7-H3*. Both qRT-PCR and western blot analyses revealed that myogenin was upregulated in all the three cell lines by transfection with siPF or siB7-H3 (Fig. 6a,b). To investigate whether upregulation of myogenin led to differentiation, we examined the expression level of myosin heavy chain (MyHC) in the ARMS cell lines. Immunofluorescence of MyHC revealed that in Rh-30 cells, transfection with siPF induced MyHC more strongly than the transfection with siB7-H3, whereas in Rh-41 cells, transfection with siB7-H3 induced MyHC more strongly than did siPF (Fig. 6c).

## Discussion

In this study, we investigated the regulatory mechanism and function of B7-H3 in PAX3-FOXO1-positive ARMS. PAX3-FOXO1 regulates the expression of many target genes^25^, and we determined that PAX3-FOXO1 also regulates B7-H3 expression in ARMS. Our data indicate that although regulated by PAX3-FOXO1, B7-H3 regulates the expression of numerous genes, some of which are also regulated by PAX3-FOXO1. Therefore, B7-H3 is considered to be an important protein downstream of PAX3-FOXO1 that contributes to aggressive characteristics of PAX3-FOXO1-positive ARMS.

Based on the GSEA results, we demonstrate that B7-H3 is associated with cell migration in ARMS, similar to that in various other cancers. However, the mechanisms by which B7-H3 regulates migration differ according to the type of cancer. For example, B7-H3 regulates CXCR4 in gastric cancer^16^, PI3K signaling in bladder and colorectal cancers^14,15^, JAK2-STAT3 signaling in hepatocellular carcinoma^28^, and MMP-2 in melanoma^29^. Our data show that B7-H3 regulates CXCR4 in ARMS. Notably, CXCR4 is also regulated by PAX3-FOXO1^30^. Previous report have shown that antibody to CXCR4 inhibit metastasis in ARMS ^31^. In addition, we found that PAX3-FOXO1 regulates the expression of STYK1, which is also associated with tumor invasion and metastasis^32,33^, although molecular targeted therapy against STYK1 is not developed. B7-H3 also regulates STYK1 expression in Rh-30 and Rh-41 cells, but not in Rh-28 cells, indicating that the effect of B7-H3 on STYK1 varies depending on the ARMS cell line.

Furthermore, we demonstrate that knockdown of *B7-H3*, as well as of *PAX3-FOXO1*, upregulates the expression of myogenin, a transcription factor needed for myogenic differentiation^34^. Although PAX3-FOXO1 inhibits myogenic differentiation in ARMS^26,35^, the effect of B7-H3 on myogenic differentiation has not been determined. Among the various functions of B7-H3 in cancer, inhibition of differentiation (not limited to myogenic differentiation) has not been reported. However, despite the observation regarding the induction of myogenin by B7-H3 knockdown in all three cell lines, the induction of further differentiation as detected by the expression of MyHC was found to vary with the cell line. Knockdown of *PAX3-FOXO1* induced the expression of MyHC more strongly than did the knockdown of *B7-H3* in Rh-30 cells, whereas *B7-H3* knockdown induced the expression of MyHC to a greater extent than did *PAX3-FOXO1* in Rh-41 cells, indicating that myogenin expression, which leads directly to increased expression of MyHC, may be dependent on the cellular context.

These results suggest that B7-H3 might be a potential therapeutic target in ARMS with PAX3-FOXO1, which itself is not yet targetable in the present clinical settings. In various cancers, B7-H3 is an attractive surface antigen for molecular targeted therapy^36^. Enoblituzumab (MGA271) is a monoclonal antibody for B7-H3 and causes antibody-dependent cell-mediated cytotoxicity (ADCC)^11^. Enoblituzumab was evaluated in a phase 1 clinical study and demonstrated acceptable tolerability in the treated patients with solid cancer (trial NCT01391143). Additionally, antibody drug conjugate (ADC) therapies and CAR T cells for B7-H3 also demonstrated outstanding results in preclinical studies and in a phase 1 clinical trial for childhood cancer^10,12,37,38^. These targeted therapies for B7-H3 might be used for treating *PAX3-FOXO1* positive ARMS that exhibits a poor prognosis with conventional intensive therapy. A phase 1 clinical trial of enoblituzumab in children and young adults with B7-H3-expressing relapsed or refractory malignant solid tumors, including ARMS, is currently being conducted (trial NCT02982941).

Our results demonstrate that B7-H3 affects cell migration and myogenic differentiation and is one of the cancer driver genes in *PAX3-FOXO1* positive ARMS. As cancer antigen escape seems less likely to occur when the cancer driver gene is a target antigen, B7-H3-targeting immunotherapy might be an attractive treatment option for *PAX3-FOXO1* positive ARMS, although we did not examine the anti-B7-H3 targeted therapy for ARMS in this study. The analysis of B7-H3 targeted therapy is warranted in the future.

This study has several limitations. First, we did not investigate the association between B7-H3 and tumor immunity in ARMS, although the GSEA indicated that gene sets related to INF-γ production and T cell-mediated cytotoxicity were inactivated by knockdown of *PAX3-FOXO1* or *B7-H3*. As B7-H3 is thought to be involved in tumor evasion from host immunity in other cancers, the relationship between B7-H3 and tumor evasion should be a focus of future studies. Second, we used only one siRNA for each gene. Therefore, we could not exclude the possibility of off-target effect by siRNA. Third, we did not investigate the function of B7-H3 in ARMS by in vivo experiments. In vivo experiments using the siRNA-treated cells would provide more evidence that strengthen our study results.

In conclusion, our findings demonstrate that PAX3-FOXO1 regulates B7-H3 expression, and PAX3-FOXO1 and B7-H3 are commonly associated with multiple pathways related to an aggressive phenotype in ARMS. In particular, PAX3-FOXO1 and B7-H3 contribute to cell migration and inhibit myogenic differentiation. This study provides evidence that B7-H3 contributes to the malignant characteristics of *PAX3-FOXO1* positive ARMS.

## Materials and methods

### Cell lines and cell culture

*PAX3-FOXO1* positive human ARMS cell lines, Rh-30, Rh-41, and Rh-28 that were kindly provided by Peter J. Houghton M (The Greehey Children's Cancer Research Institute, San Antonio, TX), as well as *PAX3-FOXO1* negative human ERMS cell line, RD that was obtained from JCRB (Japanese Collection of Research Bioresources) Cell Bank, were used in this study. The cells were maintained in RPMI 1640 medium (Nakarai Tesque, Kyoto, Japan), supplemented with 10% fetal bovine serum (FBS; Gibco, Carlsbad, CA), penicillin (100 U/mL), and streptomycin (100 µg/mL) at 37 °C in a humidified atmosphere of 5% CO_2_.

### siRNA and knockdown

siCont and an siB7-H3 were purchased from Invitrogen (Carlsbad, CA; catalog numbers AM4611 and AM16708-s37290, respectively). siPF was custom-synthesized as described previously^26^. Briefly, siPF targets the fusion sites between exon 7 of *PAX3* and exon 2 of *FOXO1*. The sense and antisense sequences for siPF were 5′-CCUCUCACCUCAGAAUUCAtt-3′ and 5′-UGAAUUCUGAGGUGAGAGGtt-3′, respectively. Transfection of cells with the siRNAs was carried out using Lipofectamine RNAiMAX (Invitrogen) according to the procedures recommended by the manufacturer. The final concentration of siRNAs was 5 nM. Six hours after transfection, the medium containing the siRNAs and Lipofectamine RNAiMAX was replaced with fresh RPMI1640 medium containing 10% FBS.

### RNA extraction, reverse transcription, and qRT-PCR

Total RNA was extracted from rhabdomyosarcoma cell lines using an RNeasy mini kit (Qiagen, Hilden, Germany) according to the manufacturer’s instructions. cDNA was synthesized using a SuperScript VILO cDNA synthesis kit (Invitrogen) according to the manufacturer’s instructions. qRT-PCR was carried out in a 7300 Real time PCR System (Applied Biosystems, Carlsbad, CA) with TB Green Premix Ex Taq II (Clontech Laboratories, Madison, WI) according to the manufacturer’s protocol. The PCR primers used in the study were as follows: for *GAPDH*: 5′-GCACCGTCAAGGCTGAGAAC-3′ (forward) and 5′-ATGGTGGTGAAGACGCCAGT-3′ (reverse); for *PAX3-FOXO1*: 5′-TCCAACCCCATGAACCCC-3′ (forward) and 5′-GCCATTTGGAAAACTGTGATCC-3′ (reverse); for *B7-H3*: 5′-CAAGGCAATGCATCCCTGAG-3′ (forward) and 5′-CTTCGAGTAGGGAGCGGC-3′ (reverse); for *MYOG*: 5′-GGACGGAGCTCACCCTGA-3′ (forward) and 5′-TTACACACCTTACACGCCCA-3′ (reverse). The levels of target mRNAs were determined using the delta delta CT method and were normalized to the expression level of GAPDH. All experiments were performed in triplicate.

### Western blot analysis

Twenty-four hours after transfection with siRNAs, the cells were lysed with RIPA buffer (Nakarai Tesque). Samples were boiled for 10 min in NuPAGE sample buffer (Invitrogen) and were separated by SDS–polyacrylamide gel electrophoresis. The proteins were subsequently transferred onto an Immobilon-P PVDF transfer membrane (Millipore, Bedford, MA). The membranes were blocked with 3% bovine albumin (Nakarai Tesque) prepared in phosphate-buffered saline with Tween 20 (PBS-T) and then incubated with one of the primary antibodies: β-actin (1:10,000, Sigma-Aldrich, St. Louis, MO), PAX3 (1:500, R&D Systems, Minneapolis, MN), Myogenin (1:500, Novus Biologicals, Centennial, CO), and B7-H3 (1:1000, Cell Signaling Technology, Danvers, MA). The membranes were then washed with PBS-T and incubated with anti-mouse or anti-rabbit secondary antibody (1:10,000, Santa Cruz Biotechnology, Santa Cruz, CA). Antibody binding was detected using an ECL prime detection system (GE Healthcare, Buckinghamshire, UK).

### Flow cytometry

Cells were harvested, washed twice with PBS, and incubated for 30 min on ice with PE-conjugated anti-human B7-H3 antibody (Biolegend, San Diego, CA). Next, the cells were analyzed on a FACS Calibur (BD Biosciences, Franklin Lakes, NJ) with the FlowJo software (Treestar, San Carlos, CA).

### Gene expression analysis

Total RNA was extracted from RH-30 cells transfected with siCont, siPF, or siB7-H3. The total RNA was used for cRNA target synthesis using a GeneChip 3′IVT PLUS Reagent Kit (Thermo Fisher Scientific, Waltham, CA). Biotin-labeled cRNA was hybridized to a GeneChip Human Genome U133 Plus 2.0 Array (Thermo Fisher Scientific). Hybridized arrays were stained and scanned with a Genechip 3000 7G Scanner (Thermo Fisher Scientific). Data were analyzed with the Affymetrix Expression Console Software 1.4.1. All microarray data are available at the Gene Expression Omnibus database under accession number GSE127703. GSEA was performed using GSEA 3.0 (Broad Institute, San Diego, CA). A group consisting of RH-30-siPF and RH-30-siB7-H3 was compared with RH-30-siCont, and gene sets at a nominal *p* value (NOM *p*-val) < 5% were considered to be significantly enriched.

### Wound healing assay

After transfection of cells with siRNAs, the cell layers were scratched using a pipette tip. Immediately after scratching (0 h), the plates were photographed and the wound area was measured using the ImageJ software and defined as the original area (100%). After 24 h of scratching, the plates were photographed again. In addition, the area of the healed wound was measured and presented as a percentage of the original area. All assays were performed in triplicate.

### Transwell migration assay

At 24 h of transfection of cells with the siRNAs, each cell line was resuspended in serum-free RPMI medium. Twenty-four well-sized transwell plates with an 8-µm pore size (Corning, Corning, NY) were used for this assay. First, 1.0 × 10^5^ cells suspended in 100 µL of serum-free medium were seeded on each insert, and 650 µL of RPMI medium with 10% FBS was added to the receiver plates. The plates were incubated for 24 h, and cells migrating onto the receiver plates were quantified as relative light units (RLU) using the Cell Titer Glo luminescent cell viability assay (Promega, Heidelberg, Germany) according to the procedures recommended by the manufacturer. All the assays were performed in triplicate.

### Immunofluorescence

At 24 h of transfection of cells with the siRNAs, Rh-30 and Rh-41 cells were resuspended in serum-free RPMI medium. After 7 days of resuspension, cells were fixed with 4% paraformaldehyde and permeabilized with 0.1% Triton-X100. Next, the cells were washed and incubated with anti-MyHC antibody (Sigma-Aldrich) overnight (1:350), rinsed with PBS, and incubated with Alexa Fluor 488 goat anti-mouse IgG (Life Technologies, Carlsbad, CA) for 1 h (1:200). Nucleic acids were stained with Hoechst, using the NucBlue Live Cell Stain Ready Probes Reagent (Invitrogen). A BZ-X800 confocal microscope (KEYENCE, Osaka, Japan) was used for observation.

### Statistical analysis

The results are shown as mean ± SD of three independent experiments. Statistical analysis was performed using the two-tailed Student’s *t* test. A *p* value < 0.05 was considered statistically significant.

## Supplementary Information

Supplementary Information.

## Abbreviations

ARMS: Alveolar rhabdomyosarcoma
CAR: Chimeric antigen receptor
ERMS: Embryonal rhabdomyosarcoma
GSEA: Gene set enrichment analysis
qRT-PCR: Quantitative real time polymerase chain reaction
RMS: Rhabdomyosarcoma
siB7-H3: SiRNA targeting *B7-H3*
siCont: Control siRNA
siPF: SiRNA targeting *PAX3-FOXO1*
