# Supplementary material for: Reduced B7-H3 expression by PAX3-FOXO1 knockdown inhibits cellular motility and promotes myogenic differentiation in alveolar rhabdomyosarcoma
Source: Sci Rep. 2021 Sep 22;11:18802. doi: 10.1038/s41598-021-98322-z (PMC8458399; doi:10.1038/s41598-021-98322-z)

Supplemental Figure 1. Full length blots of Western blot analysis.
The blots shown in Figure 6 (b) were cropped from these full length blots.

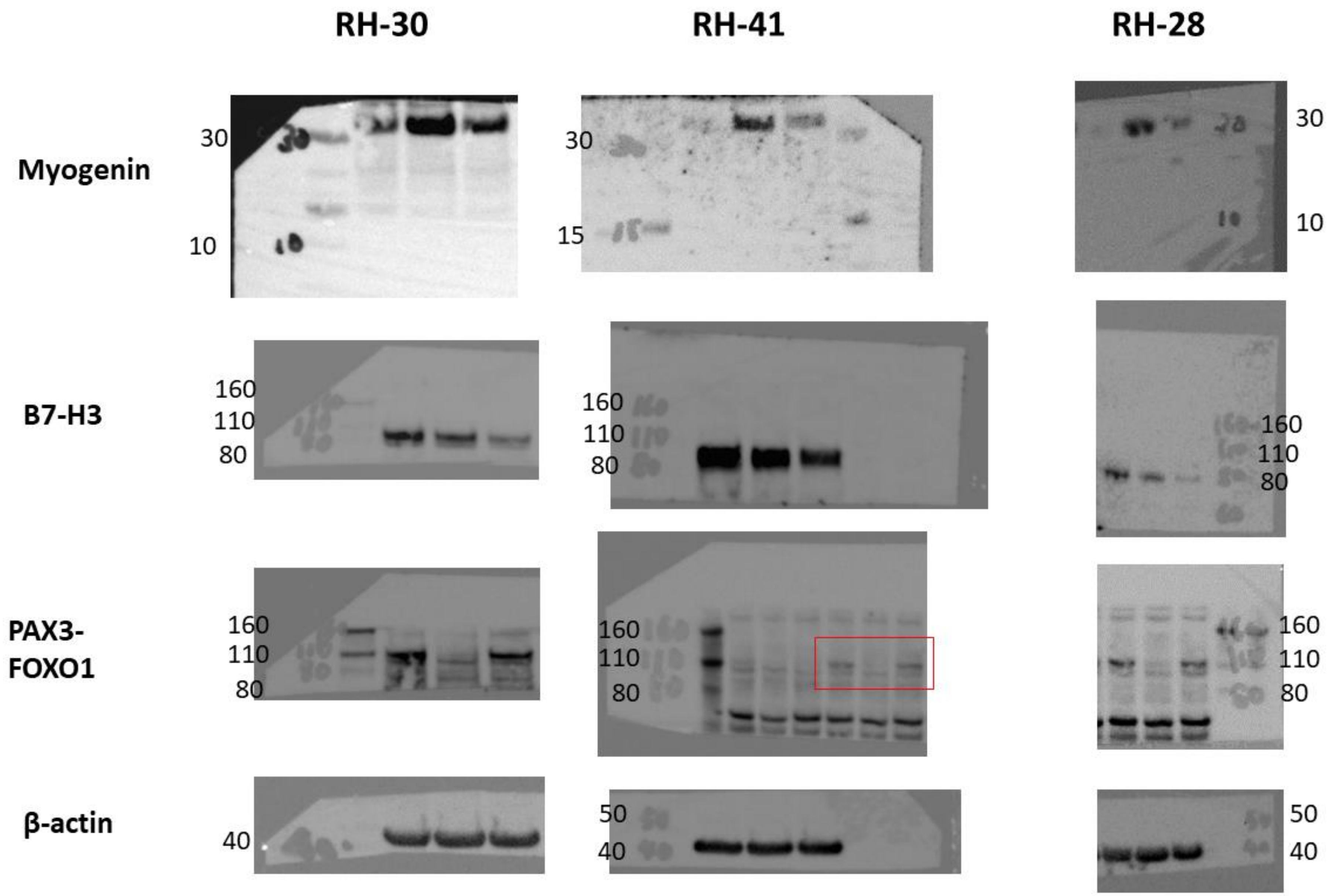

Supplement: Supplementary file 1 — Supplementary Information. [file 41598_2021_98322_MOESM1_ESM.pdf]
